# C‐reactive protein: An independent predictor for dedifferentiated chondrosarcoma

**DOI:** 10.1002/jor.24030

**Published:** 2018-05-24

**Authors:** Elena Nemecek, Philipp Theodor Funovics, Gerhard Martin Hobusch, Susanna Lang, Madeleine Willegger, Florian Sevelda, Thomas Brodowicz, Christoph Stihsen, Reinhard Windhager, Joannis Panotopoulos

**Affiliations:** ^1^ Department of Orthopaedics and Traumatology Clinical Division of Orthopaedics, Medical University of Vienna Währinger Gürtel 18‐20, A‐1090 Vienna Austria; ^2^ Clinical Institute of Pathology Medical University of Vienna Vienna Austria; ^3^ Department of Medicine I, Clinical Division of Oncology Medical University of Vienna Vienna Austria

**Keywords:** dedifferentiated chondrosarcoma, prognosis, CRP

## Abstract

Dedifferentiated chondrosarcoma is a rare primary bone malignancy with a very poor prognosis. The aim of the study was to identify pretreatment serum markers as prognostic factors for the overall survival (OS) of patients with dedifferentiated chondrosarcoma. We retrospectively reviewed 33 patients with histologically confirmed dedifferentiated chondrosarcoma treated at our department from 1977 to 2015. Kaplan‐Meier estimation, uni‐ and multivariable Cox proportional hazard model were performed to evaluate the association between serum markers such as the C‐reactive protein and OS. In univariable analysis, CRP was strongly associated with OS (HR 1.35; 95%CI 1.13–1.61; *p* = 0.001). This association prevailed after adjustment for AJCC tumor stage (HR 1.31; 95%CI 1.02–1.57; *p* = 0.031) in multivariable analysis. In conclusion, our data gave evidence that baseline CRP is an independent predictor for OS in patients with dedifferentiated chondrosarcoma. CRP could be exploited for the clinical prediction of this disease in the future. © 2018 The Authors. *Journal of Orthopaedic Research*® Published by Wiley Periodicals, Inc. on behalf of Orthopaedic Research Society. J Orthop Res 36:2797–2801, 2018.

Dedifferentiated chondrosarcoma is a rare primary bone malignancy with a very poor prognosis. First described by Dahlin and Bebout in 1971,^1^ it is characterized by high‐grade, non‐cartilaginous sarcoma adjacent to a low‐grade or intermediate chondrosarcoma and makes about 10% of all chondrosarcomas.^2^

The surgical resection with wide or radical margins is considered as standard and most important therapy option, though the prognosis is poor with 5‐year survival rates mostly under 20%.^2^ The National Comprehensive Cancer Network guidelines suggest that the dedifferentiated chondrosarcoma should be treated like the osteosarcoma.^3^

For risk estimation, several prognostic markers such as location, pathologic fracture, age > 60, size, metastasis at diagnosis, have been described previously.^2^, ^4^

In recent studies, the concept of the involvement of systemic inflammation in cancer progression and metastasis has been postulated.^5^, ^6^ Specifically, elevated pre‐operative CRP as marker of systemic inflammatory response has been found to be associated with decreased overall survival in osteosarcoma, chordoma, and soft tissue sarcoma.^5^, ^7^, ^8^, ^9^

To the best of our knowledge, no previous reports included the C‐reactive protein (CRP) as prognostic factor in survival of dedifferentiated chondrosarcoma. For this purpose, we set up a cohort study to analyze the association of pre‐operative serum CRP with overall survival in patients with dedifferentiated chondrosarcoma.

## METHODS

From 1977 to 2015, a total of 33 patients with histological confirmed dedifferentiated chondrosarcoma underwent surgery at the Department of Orthopedic Surgery, Medical University of Vienna and were followed‐up until December 2017. No previous surgical intervention to the sarcoma site has been performed at an outside facility.

All patients were followed up at our department every 3 months during the first 3 years, every 6 months in years 4 and 5, and in 12‐month intervals thereafter. Post‐operative surveillance consisted of clinical examination, computed tomography of the thorax, abdominal ultrasound, and local magnetic resonance imaging.

Complete clinical pathological data were available for all 33 included patients and were retrospectively collected from medical reports (sex, age, tumor site, histology, tumor size, resection margins, tumor stage, laboratory parameters, adjuvant radiotherapy, adjuvant chemotherapy, local recurrence). Laboratory data from routine investigations were obtained 1–14 days prior to first surgical treatment. Pre‐operative in‐house assessment of C‐reactive protein was available in 27 patients. CRP measurements were performed as a part of pre‐operative clinical routine by a latex‐enhanced immunoturbodimetric test.

Histological diagnoses were classified according to the current WHO classification for soft tissue and bone tumors (according to Fletcher et al.^10^), and reconfirmed by an experienced pathologist specialized in bone sarcoma at our hospital. Resection margins were classified according to Enneking et al.^11^ Tumor‐stage was generated according to American Joint Committee on Cancer (AJCC) criteria.^12^

In accordance to previous reports we converted tumor size to a categorical variable (≤8 cm, >8 cm) based on the AJCC criteria.^12^ Primary tumor location was categorized to upper extremity, lower extremity, axial (pelvic bones, sacrum, spine), or chest wall (rib, sternum, clavicle), patient age was categorized to (< ≥ 60 years) as previously reported by Strotman et al.^2^

The study was approved by the local ethics committee of the Medical University of Vienna (EC‐number 767/2008).

## LEVEL OF EVIDENCE III (RETROSPECTIVE COHORT STUDY)

### Statistical Analysis

All statistical analyses and graphical visualization were performed using IBM SPSS Statistics, Version 24, SPSS Inc, Chicago, IL. Continuous data were summarized using mean values, medians and ranges, and categorical data by absolute frequencies and percentages. Correlations were calculated by the Spearmann's correlation coefficient. We defined the day of first diagnosis as baseline, and considered death from any cause as endpoint (overall survival), which in our study could carefully be considered as equal of death of disease. Follow‐up time was defined as the time from index surgery to death or last known alive. Survival probabilities were calculated with the Kaplan–Meier product limit estimator. For this purpose, serum CRP levels were categorized into <1 and ≥1 mg/dl. To compare the survivor functions of two or more patient groups, we applied the log‐rank test. Uni‐ and multivariable Cox proportional hazards regression models were fitted to evaluate the association between baseline variables and survival. The multivariable model was calculated with the co‐variable AJCC tumor stage. *p*‐Values <0.05 were considered to indicate statistical significance.

## RESULTS

**Figure 1:**
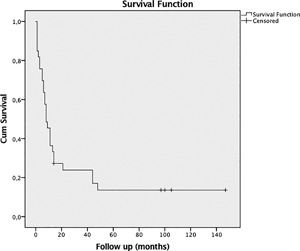
Cumulative survival and follow‐up in months is shown in this Kaplan Meier estimates. The overall survival was 63.6% after 6 months, 36.4% after 1 year, 23.9% after 2, and 13.6% after 5 years.

Seventeen patients of our study population were female (51.5%) and 16 were male (48.5%). The mean age was 62.2 years (SD 14.43; range, 22–90) (for demographic data, see Table 1). The primary tumor was located as follows: Lower extremity in 13 cases (39.4%), upper extremity in 2 (6.1%), axial location in 15 (45.5%), and chest wall in three cases (9.1%). The overall survival was 63.6% after 6 months, 36.4% after 1 year, 23.9% after 2 years, and 13.6% after 5 years (see Fig. 1).

**Table 1:** Demographic Data of the Patients Were Listed

Age (years)	62.2 (22–90)
Metastasis at diagnosis	
None	19 (57.6%)
Bone	2 (6.1%)
Lung	4 (12.1%)
Lung/visceral	4 (12.1%)
NA*	4 (12.1%)
Sex	
Male	16 (48.5%)
Female	17 (51.5%)
Tumor size	
≥8 cm	19 (57.6%)
<8 cm	13 (39.4%)
NA*	1 (3%)
Location	
Lower extremity	13 (39.4%)
Upper extremity	2 (6.1%)
Axial location	15 (45.5%)
Chest wall	3 (9.1%)
Surgery	
No surgery	1 (3%)
Non‐amputation	29 (87.9%)
Amputation	3 (9.1%)
Resection margins	
Wide/radical	26 (78.8%)
Marginal	4 (12.1%)
Intralesional	2 (6.1%)
NA*	1 (3%)
Pathological fracture	
No path fracture	23 (69.7%)
Path fracture	9 (27.3%)
NA*	1 (3%)
Local recurrence	
Local recurrence	7 (21.2%)
No local recurrence	24 (72.7%)
NA*	2 (6.1%)
AJCC tumor stage	
Stage I	0 (0%)
Stage II	19 (57.6%)
Stage III	2 (6.1%)
Stage IV	8 (24.2%)
NA*	4 (12.1%)

Patients were grouped according to AJCC tumor stage (*NA, not available).

The mean CRP at baseline was 1.85 mg/dl (SD 2.37; range, 0.18–9.34) (median 1.15), the mean fibrinogen was 484 mg/dl (SD 129; range, 297–838). We observed a strong correlation between CRP and fibrinogen (Spearmann's rho = 0.85, *p* < 0.0001).

In univariable analysis, CRP (HR 1.35; 95%CI 1.13–1.61; *p* = 0.001) was strongly associated with overall survival (Table 2). Metastasis at baseline (HR 1.26; 95%CI; 1.01–1.57; *p* = 0.045) and AJCC Tumor Stage (HR 1.82; 95%CI, 1.17–2.84; *p* = 0.008) as categorical variable were associated with decreased overall survival. Primary tumor location, resection margins, chemotherapy, pathological fractures were not associated with overall survival (Table 2).

**Table 2:** Univariable Associations of Above Listed Variables Such As Serum Markers, Treatment, Tumor Stage, and Location as well as AJCC With Overall Survival

	Analysis of Overall Survival (OS)		
Variable	HR	95%CI	*p*‐Value
CRP	1.35	1.13–1.61	0.001*
Hemoglobin	0.59	0.37–0.96	0.031*
Fibrinogen	1.01	1.00–1.01	0.008*
Age	1.03	0.99–1.06	0.61
Size	0.53	0.36–1.21	0.13
Metastasis	1.26	1.01–1.57	0.045*
Chemotherapy	0.91	0.42–1.92	0.79
Location	0.89	0.63–1.26	0.51
Margins	1.21	0.64–2.28	0.55
AJCC	1.82	1.17–2.84	0.008*
Albumin	0.85	0.76–0.96	0.006*
Path. fracture	0.46	0.20–1.08	0.74

A confidence interval of 95% was set, *p*‐values <0.05 are marked with *.

In multivariable analysis, CRP remained a strong predictor for overall survival when adjusted for AJCC tumor stage (HR 1.31 95%CI 1.02–1.57; *p* = 0.031) (see Table 3).

**Table 3:** Multivariable Associations With Overall Survival Adjusted for AJCC Tumor Stage

	Analysis of Overall Survival (OS) Adjusted for AJCC Tumor Stage		
Variable	HR	95%CI	*p*‐Value
CRP	1.31	1.02–1.57	0.031*
Albumin	0.87	0.75–1.00	0.043*
Hemoglobin	0.66	0.41–1.08	0.1
Fibrinogen	1.00	1.00–1.01	0.018*

*p*‐Values <0.05 are marked with *.

**Figure 2:**
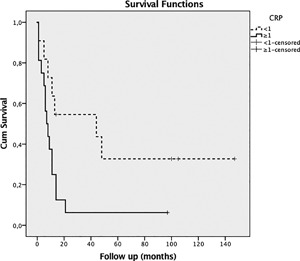
The survival rate for patients with pre‐operative CRP levels <1 mg/dl (‐‐‐, curve) was significantly higher with 36.4% when compared to the survival rate of 6.3% for patients with CRP levels 1.0 mg/dl (‐, curve).

**Figure 3:**
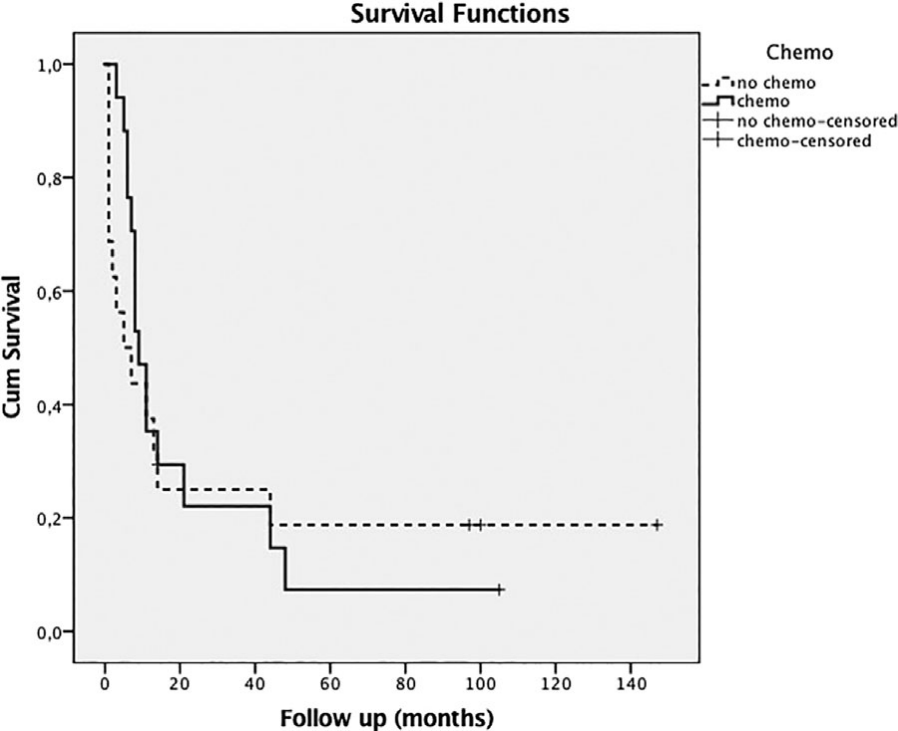
When looking at cumulative survival, there was no statistically significant difference in patients with or without chemotherapy (‐, chemotherapy; ‐‐‐, no chemotherapy).

Patients with pre‐operative CRP levels ≥1.0 mg/dl had a lower survival rate than patients with CRP levels <1 mg/dl (Log Rank *p* = 0.026) (survival rates of 6.3% vs. 36.4%) (see Fig. 2). There was no difference between patients who were treated by surgery or chemotherapy and surgery alone (Log Rank *p* = 0.79; see Fig. 3).

## DISCUSSION

Dedifferentiated chondrosarcoma has a very poor prognosis. The state of the art treatment consists of the wide resection of the tumor. Survival rates did not improve over the last decades despite aggressive resection, systemic treatment with chemotherapy and advanced diagnostic techniques.^2^ Our aim was to find pre‐treatment serum markers as independent factors for estimation of survival in chondrosarcoma patients. Several prognostic factors have been described in previous reports: Pelvic location, increased age, metastasis at diagnosis,^4^ pathological fracture,^13^ advanced tumor stage (AJCC III or IV), and size (tumors larger than 8 cm)^2^ and inadequate resection margins.^14^

To the best of our knowledge no previous report aimed to define the C‐reactive protein or any acute phase protein as prognostic factor. Over the last years, the involvement of systemic inflammation in cancer progression and metastasis has been postulated. Cancer cells may interact directly and indirectly with host inflammatory cells.^6^, ^9^, ^15^ Especially the C‐reactive protein (an acute phase protein synthesized by the liver) as a marker of systemic inflammatory response has been found to be associated with decreased survival rates in different malignancies.^5^, ^7^, ^8^, ^16^, ^17^

Our observation of an association between elevated CRP and worse overall survival is consistent with the previous findings in other malignancies. In the present study, CRP was strongly correlated to fibrinogen and both were strongly associated with decreased overall survival CRP remained significant after adjustment with AJCC tumor stage in a multivariable regression model. We chose AJCC tumor stage as adjustment in the multivariable model because it captures tumor specific information in only one variable: Tumor size, bone, lung and visceral metastasis. We did the analysis for each AJCC stage separately and performed furthermore the analysis with AJCC stage II versus stage III/IV and stage II/III versus stage IV—in every analysis, the CRP remained an independent prognostic factor (data not shown, can be provided upon request).

If we consider both, pre‐operative CRP and fibrinogen, as surrogates for inflammatory response, our results could be explained by the concept of systemic inflammation in cancer.

Though the National Comprehensive Cancer Network guidelines suggest that the dedifferentiated chondrosarcoma should be treated like the osteosarcoma, Dickey et al.^18^ did not find any difference in overall survival comparing surgery alone to surgery and chemotherapy (*p* = 0.54). In a multicenter study conducted by Grimer et al.,^4^ no difference in overall survival was observed in patients under 60 years without metastasis at diagnosis whether they received chemotherapy or not (*p* = 0.09). The authors have no sufficient explanation for the lack of benefit of systemic treatment.

Our data confirm the poor outcome with an observed 5‐year survival rate of 13.6% and the ineffectiveness of chemotherapy (Log Rank *p* = 0.79).

We are aware of the limitations of the present study (retrospective design, sample size); a strong point of our report is that in contrast to Strotman et al.^2^ and Grimer et al.^4^ we were able to reconfirm the histological diagnosis by an experienced bone tumor pathologist and co‐author of the present report (SL). Another limitation could be found in the fact, that the C‐reactive protein is influenced by other factors than inflammation, for example pathologic fractures. In our study, however, CRP remained significant after adjustment for the co‐variable “pathological fracture.” Moreover, the CRP remained significant after adjustment for the co‐variable “size” (data not shown, can be provided upon request).

We can carefully speculate that the pre‐operative CRP is an independent predictor for survival in dedifferentiated chondrosarcoma.

## CONCLUSION

Our data gave evidence that baseline CRP is an independent predictor for OS in patients with dedifferentiated chondrosarcoma. CRP could be exploited for the clinical prediction of this disease in the future.

## AUTHORS' CONTRIBUTIONS

Elena Nemecek, Joannis Panotopoulos, Thomas Brodowicz, Gerhard Martin Hobusch, and Susanna Lang performed research design, analysis and interpretation of data, and drafted the manuscript. Madeleine Willegger and Florian Sevelda performed acquisition of data. Philipp Theodor Funovics, Reinhard Windhager, and Christoph Stihsen revised the paper critically. All authors have read and approved the submitted and final version of the paper.
